# Suppressing BCL-XL increased the high dose androgens therapeutic effect to better induce the Enzalutamide-resistant prostate cancer autophagic cell death

**DOI:** 10.1038/s41419-020-03321-z

**Published:** 2021-01-11

**Authors:** Zhendong Xiang, Yin Sun, Bosen You, Meng Zhang, Chiping Huang, Junfeng Yu, Xiangyun You, Denglong Wu, Chawnshang Chang

**Affiliations:** 1 Department of Urology, Tongji Hospital, Tongji University School of Medicine, 200065 Shanghai, China; 2 George Whipple Lab for Cancer Research, Departments of Pathology, Urology, Radiation Oncology and The Wilmot Cancer Center, University of Rochester Medical Center, Rochester, NY 14642 USA; 3 Department of Urology, The People’s Hospital of China Three Gorges University, The First People’s Hospital of Yichang, 443003 Yichang, China; 4 Department of Urology, The 4th Hospital of Harbin Medical University, 150000 Harbin, China; 5 Sex Hormone Research Center and Department of Urology, China Medical University/Hospital, Taichung, 404 Taiwan

**Keywords:** Autophagy, Prostate cancer

## Abstract

Most patients with advanced prostate cancer (PCa) initially respond well to androgen deprivation therapy (ADT) with antiandrogens, but most of them eventually become resistant to ADT. Here, we found that the antiandrogen Enzalutamide-resistant (EnzR) PCa cells can be suppressed by hyper-physiological doses of the androgen DHT. Mechanism dissection indicates that while androgens/androgen receptor (AR) can decrease BCL-2 expression to induce cell death, yet they can also simultaneously increase anti-apoptosis BCL-XL protein expression *via* decreasing its potential E3 ubiquitin ligase, PARK2, through transcriptionally increasing the miR-493-3p expression to target PARK2. Thus, targeting the high dose DHT/AR/miR-493-3p/PARK2/BCL-XL signaling with BCL-XL-shRNA can increase high-dose-DHT effect to better suppress EnzR cell growth *via* increasing the autophagic cell death. A preclinical study using in vivo mouse model also validated that suppressing BCL-XL led to enhance high dose DHT effect to induce PCa cell death. The success of human clinical trials in the future may help us to develop a novel therapy using high dose androgens to better suppress CRPC progression.

## Introduction

For non-metastatic castration-resistant prostate cancer (CRPC) patients, the antiandrogen Enzalutamide (Enz) can reduce the risk of metastasis or death by 71% and is associated with significantly longer progression-free and overall survival than standard care in men with metastatic, hormone-sensitive prostate cancer receiving testosterone suppression^1–3^. But most patients will eventually develop Enz resistance with limited therapeutic options, mainly cytotoxic chemotherapy^4–6^. Continuous ADT and chemotherapy reduce the quality of life of patients.

Bipolar androgen therapy is an emerging therapeutic approach to achieve the inhibition of advanced PCa through a cycle of androgen receptor (AR) inhibition followed by AR activation with a combination of other drugs^7,8^, and other studies indicated that high-dose-of androgen might have a good tolerance in some selective CRPC patients^9,10^. This treatment can not only improve the quality of life of patients but also restore the sensitivity of some CRPC patients to the androgen deprivation therapy (ADT) treatment. The mechanism of high dose androgen inhibiting PCa is still not clear. Several hypotheses have been proposed for the efficacy of bipolar androgen therapy^7,9,10^. One suggests that androgen-induced AR nuclear translocation results in DNA damage and cell proliferation inhibition or apoptosis^11^. Another suggests that androgen-activated AR serves as a licensing factor for DNA replication to block cell proliferation^12^, in addition to its ability to induce the expression of CDK inhibitor p27^Kip1^ ^13,14^. Earlier studies indicating that DHT could suppress the expression of BCL-2, one of the key anti-apoptotic proteins, in prostate and breast cancer^15–18^. Other studies have shown that androgen can cause cell G0/G1 arrest in LNCaP and VCaP cells under the conditions of slightly hypotonic growth medium (LD-hypo)^8^. Understanding the mechanisms underlying bipolar androgen therapy will help design therapies to enhance its efficacy while providing precision medicine for patients who can benefit from this treatment.

We report here that androgens can induce the autophagic cell death through altering the BCL-2/Beclin-1 axis, which is mitigated by an increase of BCL-XL caused by androgen-induced microRNA (miR), miR-493-3p to suppress its E3 ubiquitin ligase PARK2. Therefore, reducing the expression of BCL-XL can increase the inhibitory effect of DHT to better suppress the growth of Enzalutamide-resistant (EnzR) cells.

## Results

### A high dose of DHT can function *via* AR to suppress the growth of EnzR PCa cells

Bipolar androgen therapy for PCa patients and cells has been tested at the stage of castration-resistant prostate cancer (CRPC)^7,10^. To further study if DHT can also inhibit PCa cells that are resistant to Enz, the most recent and potent regiment of ADT^19^, we generated CRPC Enz-sensitive (EnzS) cells, C4-2 and C4-2B with continuous treatment of Enz to yield EnzR PCa cells. We treated EnzS-C4-2/EnzR-C4-2, EnzS-C4-2B/EnzR-C4-2B, and EnzS-LNCAP/EnzR-LNCAP pairs of cell lines with different concentrations of DHT and found that cell growth was less or not inhibited at physiological concentrations of DHT for parental EnzS cells.

**Fig. 1:**
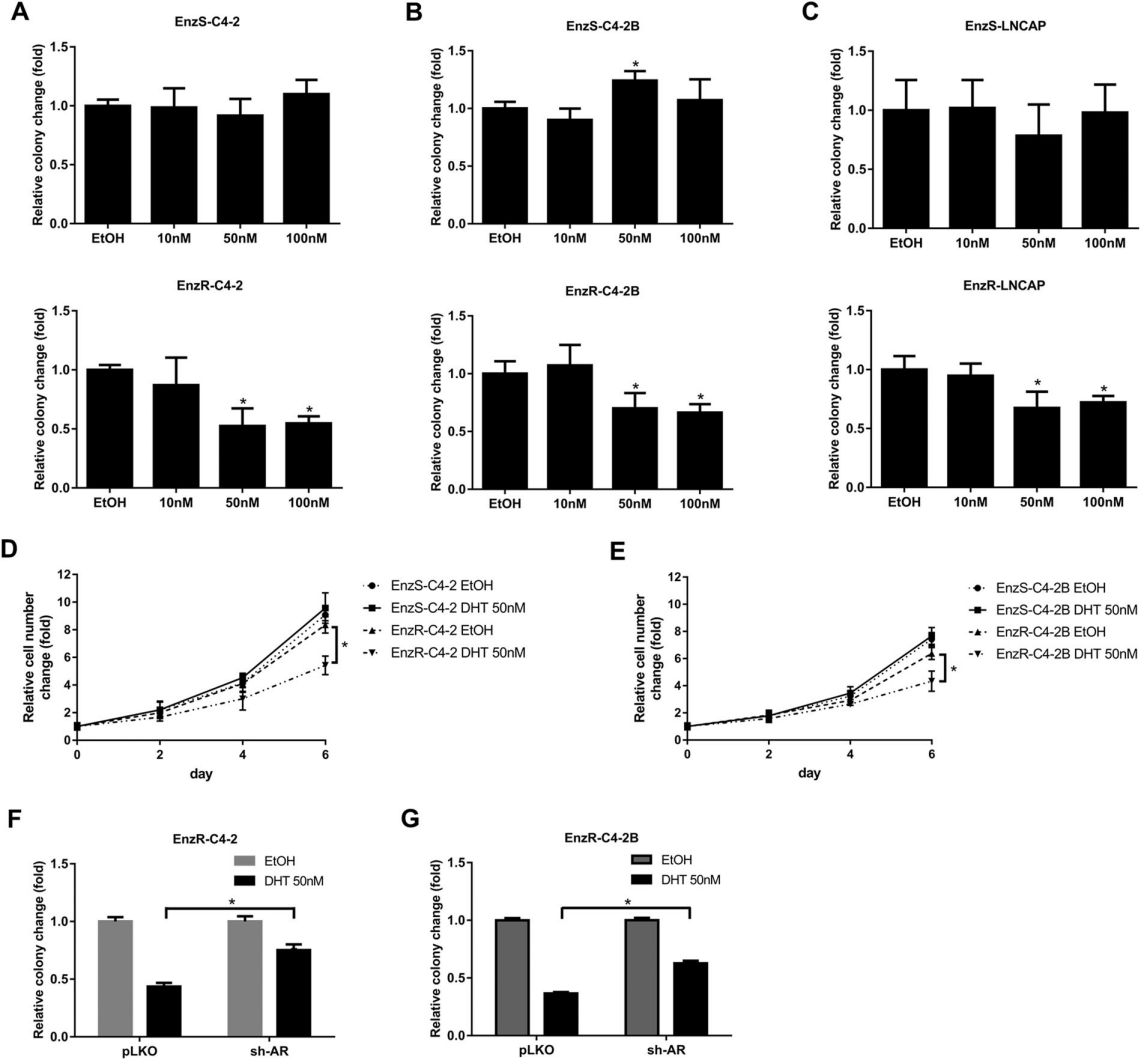
DHT can inhibit CRPC cell growth. **A**–**C** Cell colony assays were performed to show EnzS-C4-2 (**A**), EnzS-C4-2B (**B**), and EnzS-LNCAP (**C**) and Enzalutamide-resistance (EnzR) cell growth with different concentrations of DHT. **D–E** Cell counting assay to show cell proliferation in EtOH or 50 nM DHT groups. **F–G** Cell growth with 50 nM DHT when sh-AR in C4-2 and C4-2B cell line. Data are presented as means ± SD. *p < 0.05 by *t*-test for two groups or ANOVA for more than two groups.

As DHT treatment can increase energy metabolism of cells to increase formazan formation in the MTT assay (Fig. S1A), therefore MTT assay is not the best assay for measuring cell proliferation under the influence of DHT, we instead applied the colony-formation assay to study the cell proliferation. Interestingly, we found that the growth of EnzR-C4-2, EnzR-C4-2B, and EnzR-LNCAP cells were all significantly inhibited when DHT was 50 nM or higher (Fig. 1A–C). Similar effects were obtained when we replaced, DHT with Testosterone (Fig. S1B).

In addition, we found that 50 nM DHT had no inhibitory effect on the cell growth with hormone-sensitive PCa cell lines (Fig. 1A–C), or AR-variant (ARv7) expressing CWR22Rv1, and AR-negative PC3 and DU-145 cell lines (Fig. S1C).

Importantly, we also obtained the similar results when we replaced the colony-formation assay with direct counting of the cell number (Fig. 1D–E).

To implicate the AR protein in mediating this DHT suppression effect, we decreased the expression of AR in EnzR-C4-2 and EnzR-C4-2B cell lines by AR-shRNA, and results revealed that, compared with the control group, the DHT effect was decreased in the cells with a lower AR expression (Fig. 1F–G).

Together, results from Fig. 1A–G suggest high dose DHT may function *via* AR to suppress the growth of EnzR PCa cells.

### Modulating BCL-2 expression led to alter the DHT inhibition effect on the EnzR PCa cell growth

**Fig. 2:**
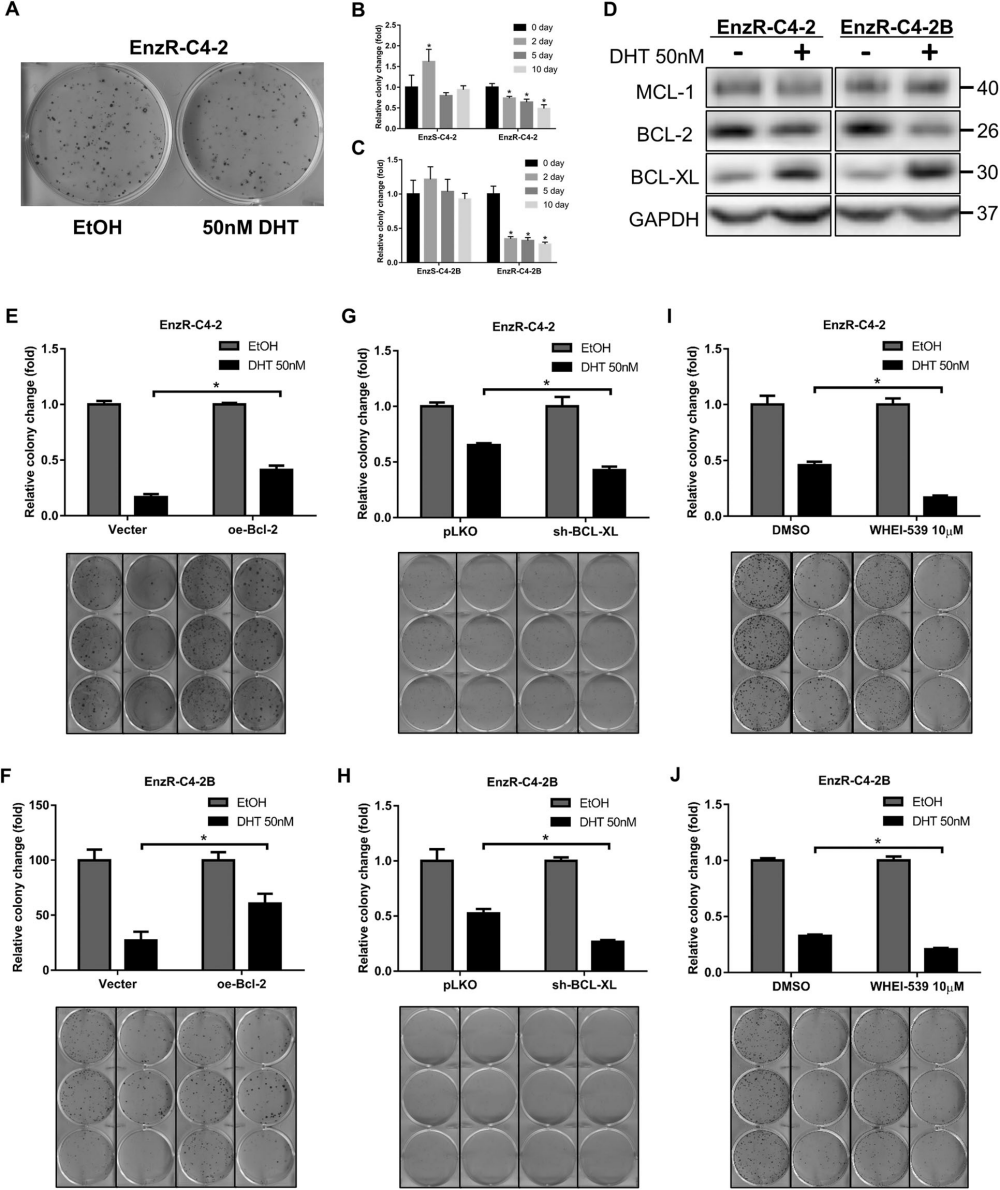
Blocking BCL-XL can enhance cell inhibition by DHT. **A** Cell colony assay shows that cell colony number changs, but size does not change. **B**–**C** Cell colony assay shows EnzR cells were treated with DHT 50 nM for 2–10 days followed by changing to regular medium (2 nM DHT, 10 μM Enzalutamide) for the remainder of the time period. **D** BCL-2 and BCL-XL protein expression in EnzR-C4-2 and EnzR-C4-2B cell line with 50 nM DHT. **E**–**F** Cell colony assay shows cell growth with 50 nM DHT after transduction of oe-BCL-2 in EnzR-C4-2 (**E**) and EnzR-C4-2B (**F**) cell line. **G**–**H** Cell colony assay shows that cell growth with 50 nM DHT after transduction of sh-BCL-XL in EnzR-C4-2 (**G**) and EnzR-C4-2B **(H**) cell line. **I**–**J** Cell colony assay shows cell growth with 50 nM DHT with/without 10 μM WHEI-539 in EnzR-C4-2 (**I**) and EnzR-C4-2B (**J**) cell line. Data are presented as means ± SD. *p < 0.05 was considered statistically significant by students’ *t*-test for two groups or ANOVA for more than two groups.

The growth inhibition of EnzR PCa cells by high dose DHT as demonstrated by colony formation assay in Fig. 1 could reflect DHT’s inhibition on the cell cycle, as well as enhanced cell death. As the number of cell colonies, but not the size of the cell colony (Fig. 2A), was significantly decreased in response to high dose DHT, it suggested that DHT might increase cell death rather than suppress cell proliferation/cell cycle of EnzR cells. Consistent with that, when 50 nM DHT was applied for 2 days or longer followed by removal with fresh regular media (2 nM DHT and 10 µM Enz) and continued growth for 10 days, there was a clear reduction of colony number by the DHT treatment in EnzR cells (Fig. 2B–C), reinforcing the notion that DHT could commit cells to death in as little as 2 days.

Results from western blot analysis revealed that BCL-2 was significantly reduced after adding 50 nM DHT, however, the expression of BCL-XL, a member of BCL-2 anti-apoptotic protein family, was increased but not another member, MCL1 (Fig. 2D).

To determine whether BCL-2 plays a role in the inhibition of EnzR cells by 50 nM DHT, we overexpressed BCL-2 protein in EnzR-C4-2 and EnzR-C4-2B cells through endogenous activation of its chromosomal gene by CRISPR-mediated transcription activation. We found that the inhibition effect by DHT on cells was weakened in the cells with overexpressed BCL-2 (Fig. 2E–F). In contrast, shRNA-mediated reduction of BCL-XL expression in EnzR-C4-2 and EnzR-C4-2B cells rendered them more inhibited by a high dose DHT (Figs. 2G–H, S1D). We also used WHEI-539, a small molecule inhibitor of BCL-XL, to inhibit BCL-XL, and found that EnzR-C4-2 and EnzR-C4-2B cells, when treated with 10 µM WHEI-539, became more inhibited by a high dose of DHT compared to the control group (Fig. 2I–J).

Together, results from Fig. 2A–J suggest that high doses of DHT in EnzR PCa cells can simultaneously regulate BCL-2 family of proteins in opposite directions with an overall impact of more cell death as measured by colony formation assay.

### Mechanism dissection of how high dose DHT *via* AR can increase the BCL-XL expression: *via* suppressing the PARK2 expression

**Fig. 3:**
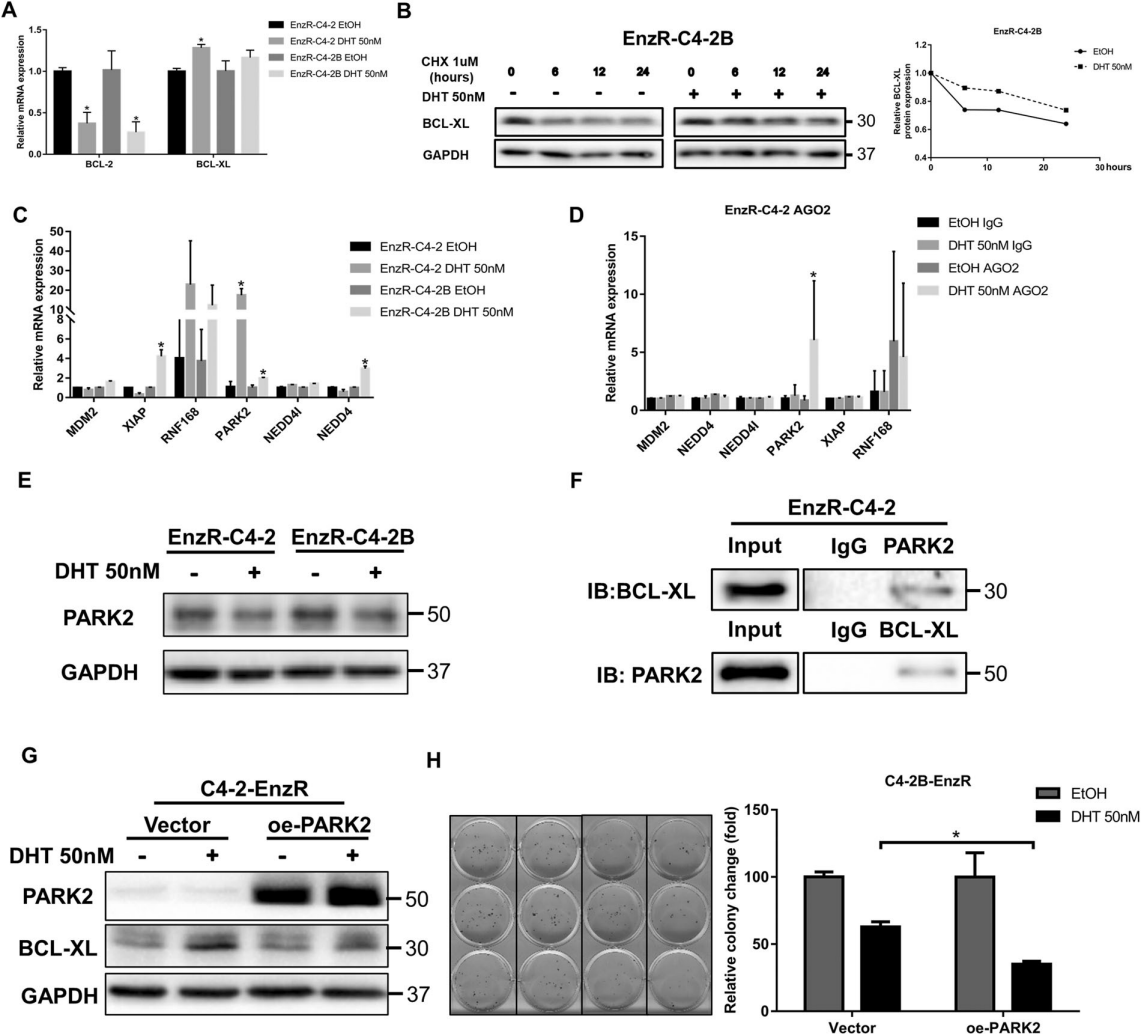
BCL-XL was regulated by PARK2. **A** BCL-2 and BCL-XL mRNA expression in EnzR-C4-2 and EnzR-C4-2B cell line treated with 50 nM DHT. **B** BCL-XL protein stability after 50 nM DHT treatment. **C** BCL-XL related E3 mRNA expression after 50 nM DHT treatment in EnzR-C4-2 cell line. **D** AGO2 IP assay to detect the E3 mRNA in AGO2 complex and results revealed that PARK2 has more miRs binding after 50 nM DHT treatment. **E** PARK2 protein expression after 50 nM DHT treatment. **F** Co-IP assay shows BCL-XL can binding to PARK2. **G** Western blotting shows BCL-XL expression after oe-PARK2 treated with 50 nM DHT. **H** Cell colony assay shows cell growth with 50 nM DHT in oe-PARK2 transduced EnzR-C4-2B cells. Data are presented as means ± SD. *p < 0.05 was considered statistically significant by students’ *t*-test for two groups.

Understanding the detailed mechanism of altering the BCL-2/BCL-XL expression by high dose DHT will likely provide novel therapeutic approaches for improved bipolar androgen therapy of PCa. We therefore examined the expression of BCL-2 and BCL-XL mRNA after high dose DHT treatment. We found that the mRNA of BCL-2 decreased significantly, consistent with the earlier report that DHT/AR can suppress BCL-2 transcription and protein expression (Fig. 3A). On the other hand, the BCL-XL mRNA was not significantly increased in response to DHT treatment (Fig. 3A). To determine whether BCL-XL increase was due to an increase of protein stability, we measured the metabolic stability of BCL-XL with the addition of cycloheximide (CHX) to block the de novo protein synthesis. The results indicated that DHT significantly increased the stability of BCL-XL in EnzR-C4-2 cells (Fig. 3B).

As ubiquitin-mediated protein degradation is responsible for regulating protein abundance for a majority of intracellular proteins while substrate-specific E3 ubiquitin ligase plus E1 and E2 are responsible for specific protein degradation through proteasome-mediated process^20,21^, we examined the mRNA expression of potential BCL-XL E3 ubiquitin ligases based on literature search and database prediction^22–26^. Six genes were identified as candidates capable of degrading BCL-XL. We detected their mRNA expression after DHT treatment and found that their mRNA expression was increased, thus not consistent with our expectation. It is possible that these proteins were regulated at the post-transcriptional level including being regulated by ncRNAs such as miRNAs. Therefore, we detected their presence in the Argonaute2 complex which represses mRNA translation by miRNA binding. This analysis led to the identification of PARK2 whose level in the AGO2 complex was significantly increased after DHT treatment (Fig. 3C–D). Consistent with this, expression of PARK2 was reduced at the protein level after DHT treatment based on western blot analysis (Fig. 3E). Indeed, endogenous BCL-XL can bind to the PARK2 protein in a co-immunoprecipitation assay (Fig. 3F). Furthermore, overexpression of PARK2 resulted in a reduction of BCL-XL in EnzR-C4-2 cells, as well as enhanced cell death by DHT (Fig. 3G–H).

Together, these results in Fig. 3A–H suggest that a high dose of DHT can decrease the PARK2 expression to increase the BCL-XL expression *via* post-transcriptional and post-translational regulatory mechanisms. The consequences of such a decrease of PARK2 expression may include an increase of the BCL-XL expression that antagonizes DHT’s effect in suppressing EnzR PCa cell growth.

### Mechanism dissection of how high dose DHT/AR can suppress PARK2 expression: *via* transcriptionally increasing the miR-493-3p expression

**Fig. 4:**
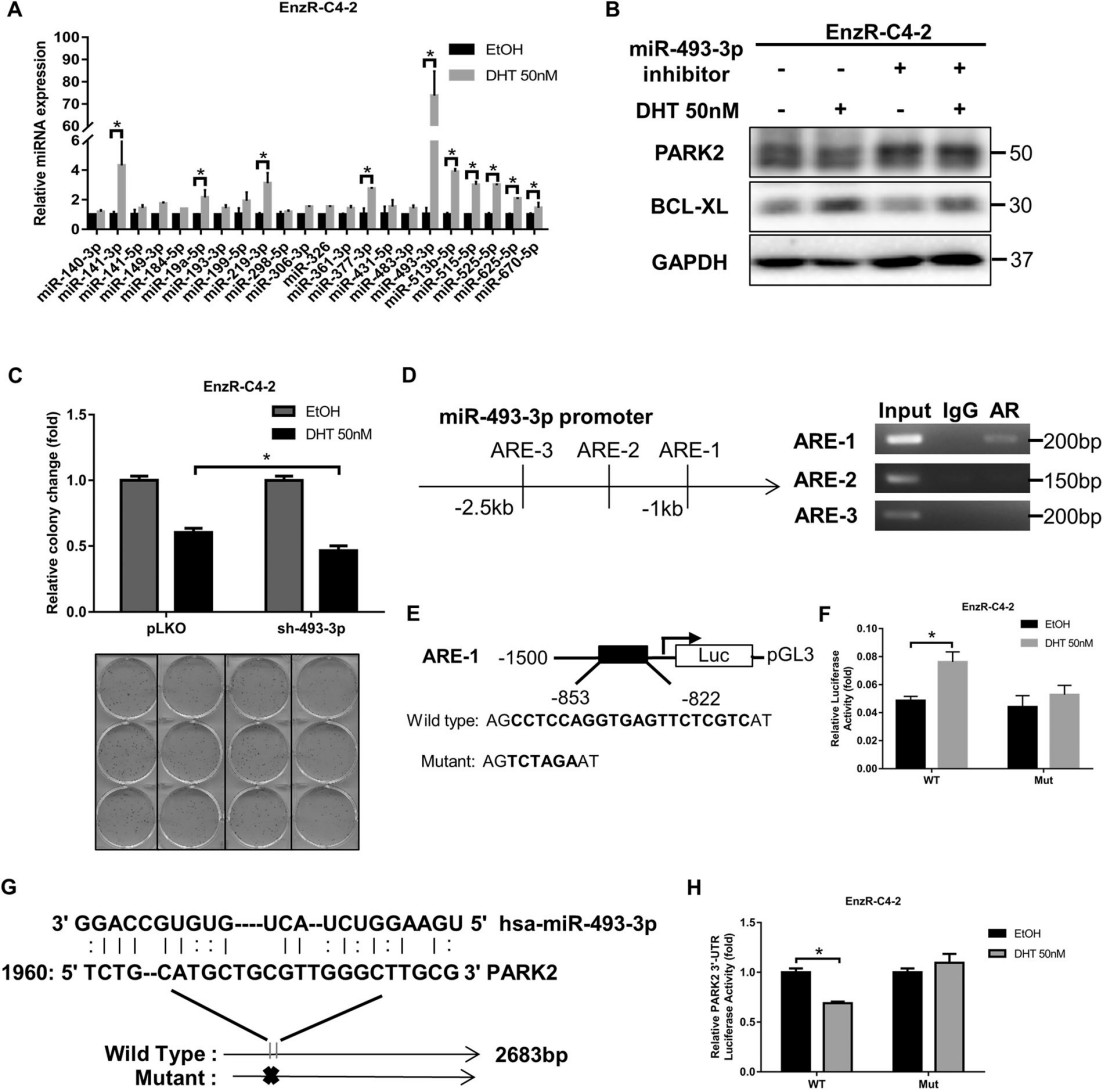
PARK2 was regulated by miR and the BCL-XL can be a therapeutic target. **A** qRT-PCR was performed to show quantification of PARK2 related miRs expression after 50 nM DHT treatment. **B** Western blotting shows BCL-XL and PARK2 expression after EnzR-C4-2 cells were treated with miR-493-3p inhibitor and 50 nM DHT. **C** Cell colony assay shows cell growth with 50 nM DHT when sh-miR-493-3p in EnzR-C4-2. **D** Potential AR response elements (AREs) were predicted on the miR-493 2.5 kb of 5’-promoter region (left). Chromatin immunoprecipitation (ChIP) binding assay was performed on EnzR-C4-2 cells (right). **E** The wild-type and mutant pGL3-miR-493 promoter-reporter constructs. **F** Luciferase activity after transfection of wild-type or mutant miR-493 promoter-reporter construct in EnzR-C4-2 cells with 50 nM DHT treatment. **G** The wild-type and mutant oe-PARK2 luciferase constructs. **H** Luciferase activity after transfection of wild-type or mutant PARK2 3’-UTR with 50 nM DHT treatment. Data are presented as means ± SD. *p < 0.05 was considered statistically significant by students’ *t*-test for two groups or ANOVA for more than two groups.

Based on the AGO2-RIP assay, it is likely that PARK2 expression can be inhibited by miRNA(s) that can be induced by DHT. To identify potential miRNAs that can target PARK2 3′UTR, we performed bioinformatic analysis from multiple databases (TargetScan, miRDB, and miRWalk), and identified 22 potential miRNAs with potential linkage to the PARK2 expression. We found that miR-493-3p expression was significantly increased after DHT treatment (Fig. 4A). Subsequently, we functionally knocked down miR-493-3p by treating with inhibitor in EnzR-C4-2 cells and found that the expression of PARK2 protein increased and the expression of BCL-XL decreased in the knockdown group (Fig. 4b). Consistent with earlier results (Fig. 3F), DHT effect was more pronounced in cells with miR-493-3p knockdown where BCL-XL expression was reduced (Fig. 4C).

To further dissect the molecular mechanism of how high dose DHT/AR can alter the miR-493-3p expression at the transcriptional level, we first applied the bioinformatic analysis to search for the potential Androgen-Response-Elements (AREs) on the miR-493 promoter using website http://jaspar.genereg.net/, and results revealed three AREs in the presumptive miR-493 promoter region (Fig. 4D). Chromatin immunoprecipitation assay with the AR antibody (ChIP) showed AR could bind to the 1st potential ARE on the miR-493 promoter region (Fig. 4D). We then constructed the wild-type and mutant pGL3-miR-493 promoter reporter plasmids (Fig. 4E), and results from luciferase reporter assay demonstrated that DHT could increase wild-type, but not mutant miR-493 promoter reporter luciferase activity (Fig. 4F).

Together, results from Fig. 4A–F suggest that high dose DHT/AR can suppress the PARK2 expression *via* transcriptionally increasing the miR-493-3p expression.

### Mechanism dissection of how high dose DHT/AR/miR-493-3p axis can suppress the PARK2 expression: *via* binding to the 3’UTR of PARK2 mRNA

Next, to verify that high dose DHT/AR/miR-493-3p axis can alter the PARK2 expression *via* binding to the 3’UTR of PARK2 mRNA, we identified potential miRNA binding sites (http://targetscan.org/) with subsequent construction of the reporter plasmids using the psicheck2 vector carrying the wild-type and mutant miRNA-target sites (Fig. 4G). As expected, the luciferase assay results revealed that adding DHT markedly decreased luciferase activity in EnzR-C4-2 cells transfected with wild-type PARK2 3′UTR but not the mutant PARK2 3′UTR (Fig. 4H).

Together, results from Fig. 4G–H suggest that high dose DHT/AR/miR-493-3p axis can suppress the PARK2 expression *via* miR-493-3p binding to the 3′UTR of PARK2 mRNA.

### High dose DHT/AR/miR-493-3p/PARK2/BCL-XL signaling suppresses EnzR cell growth *via* induction of autophagic cell death

**Fig. 5:**
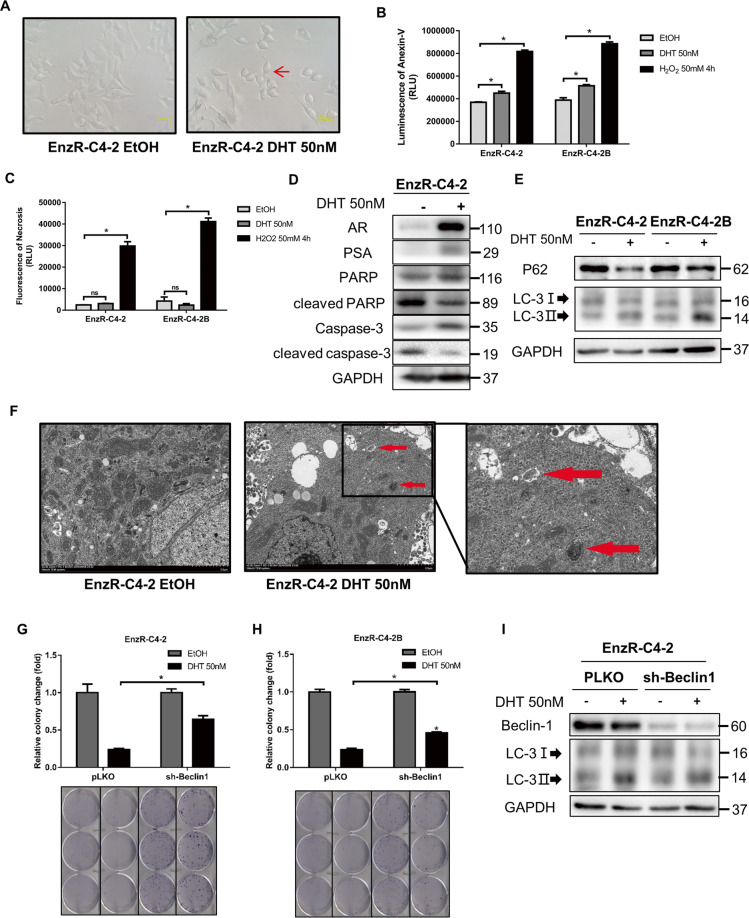
AR nuclear translocation contributed to cell autophagic cell death. **A** Morphology shows that most EnzR-C4-2 cells were balled up after 50 nM DHT treatment. **B–C** RealTime-Glo™ Annexin-V Apoptosis and Necrosis Assay were performed to show Apoptosis (**B**) and Necrosis (**C**) after 50 nM DHT or H_2_O_2_ in EnzR-C4-2 cells. **D–E** Western blotting was performed on EnzR-C4-2 cell line to show that caspase-dependent apoptosis marker is decreased (**D**) and autophagy marker is increased (**E**) with 50 nM DHT treatment. **F** Transmission electron microscopy showed the status of autophagosomes (red arrows) after 50 nM DHT treatment. **G**–**H** Cell colony assay shows cell growth with 50 nM DHT when sh-Beclin-1 in EnzR-C4-2 (**G**) and EnzR-C4-2B (**H**) cell lines. **I** Western blotting shows LC3 expression after sh-Beclin-1 with 50 nM DHT. Data are presented as means ± SD. *p < 0.05 was considered statistically significant by students’ *t*-test for two groups or ANOVA for more than two groups.

To further dissect the molecular details of how high dose DHT/AR/miR-493-3p/PARK2/BCL-XL signaling can suppress the EnzR cell growth (Fig. 5A), we focused on cell death to determine if it was mediated *via* apoptosis or necrosis. We first measured the phosphatidylserine (PS) on the outer leaflet of cell membranes as a signal for apoptosis using the Promega Annexin V Apoptosis and Necrosis Assay.

The results showed that in EnzR-C4-2 cells, after DHT treatment for 48 h, there is a significant increase of Annexin-V luminescence, indicating an increase of apoptosis (Fig. 5B). However, there was no significant increase of fluorescence thus no necrosis involved (Fig. 5C). Besides, we also measured the expression of proteins that execute the apoptosis with western blot, and results showed that Caspase-related proteins decreased after DHT treatment while autophagy-related protein LC3 increased with a decrease of P62 (Fig. 5D–E), suggesting that autophagic cell death rather than Caspase-regulated apoptosis is involved in this process. Consistent with this, when we treated EnzR-PCa cells with Z-VAD-FMK, a known inhibitor of apoptosis, the inhibitory effect of 50 nM DHT on the cells could not be reversed (Fig. S1E).

To further implicate the autophagy in DHT-induced cell death, transmission electron microscopy analysis was used to detect the vacuole-like structures of the double membranes containing mitochondria or endoplasmic reticulum in the 50 nM DHT group of EnzR-C4-2 cells (Fig. 5F). We also directly measured autophagy with the expression of GFP-LC3 in the EnzR-C4-2 cells and found that the number of cells with autophagosomes marked by GFP dots increased after DHT treatment (Fig. S1F). To strengthen the conclusion that autophagy and autophagic cell death are mediating high dose DHT’s inhibition on EnzR cells, we also examined the role of Beclin-1, the critical regulator of macro-autophagy, particularly in light of the reports that BCL-2 family can interact with Beclin-1 to regulate autophagy^27–30^. The results revealed that in Beclin-1-knocked down EnzR-C4-2 and EnzR-C4-2B cells, DHT had a less inhibitory effect compared with the control group (Fig. 5G–H). Consistent with that, LC3 II level was also decreased as a gauge of autophagy in the sh-Beclin-1 group (Fig. 5I).

Together, these results (Fig. 5A–I) suggest that high dose DHT/AR/miR-493-3p/PKRK2/BCL-XL signaling may inhibit PCa cell growth *via* Beclin-1-mediated autophagic cells death.

### Preclinical study using in vivo mouse model to test whether BCL-XL-shRNA can increase efficacy of high dose DHT to better inhibit EnzR cell growth

**Fig. 6:**
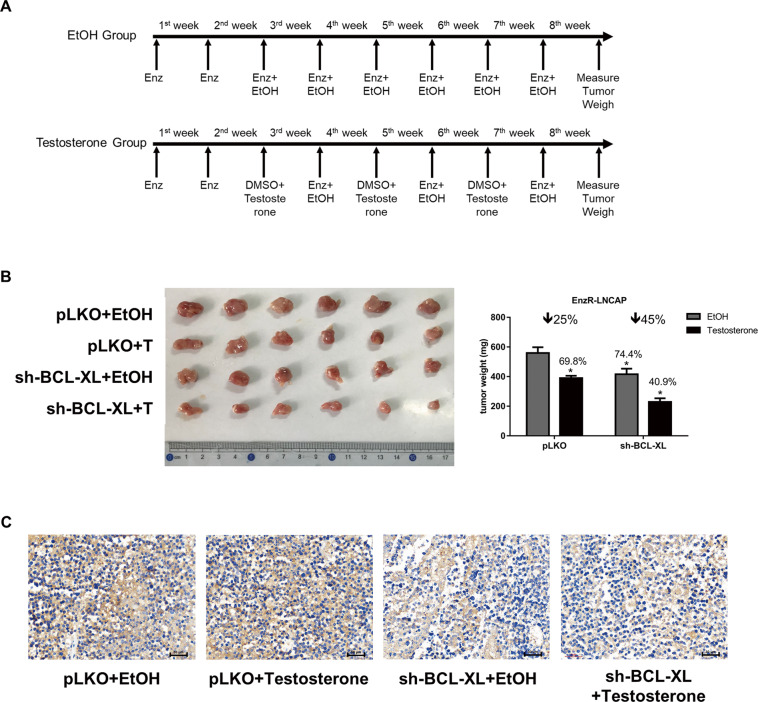
A lower BCL-XL can enhance androgen’s suppression of tumor growth in vivo. **A** Nude mouse groups were injected with EnzR-LNCAP cell suspensions (~5 × 10^6^ cells). Mice were injected with enzalutamide (10 mg/kg/week, twice weekly). Bipolar androgen therapy treatment was conducted by injection of Testosterone (200 μg/kg/week, twice weekly) or EtOH at 3th/5th/7th week. Mice were sacrificed after 8 weeks, tumors were removed and measured for studies. **B** Tumor weights were shown and represented with mean ± SD. **C** Representative IHC image of BCL-XL expression in tumor tissue samples. *p < 0.05 was considered statistically significant by students’ *t*-test compare with pLKO+EtOH groups.

To test whether targeting the BCL-XL can enhance the DHT’s suppressive effect on PCa tumor growth in a xenograft model in vivo, we generated cells with stable expression of sh-BCL-XL, as well as vector control cells and orthotopically implanted these cells in the anterior of prostates with 4 groups, 1: pLKO + EtOH; 2: pLKO + Testosterone; 3: sh-BCL-XL + EtOH; 4: sh-BCL-XL + Testosterone (Fig. 6A). After 8 weeks of treatment, we found that sh-BCL-XL + Testosterone group had the smallest tumor size (Fig. 6B–C).

**Fig. 7:**
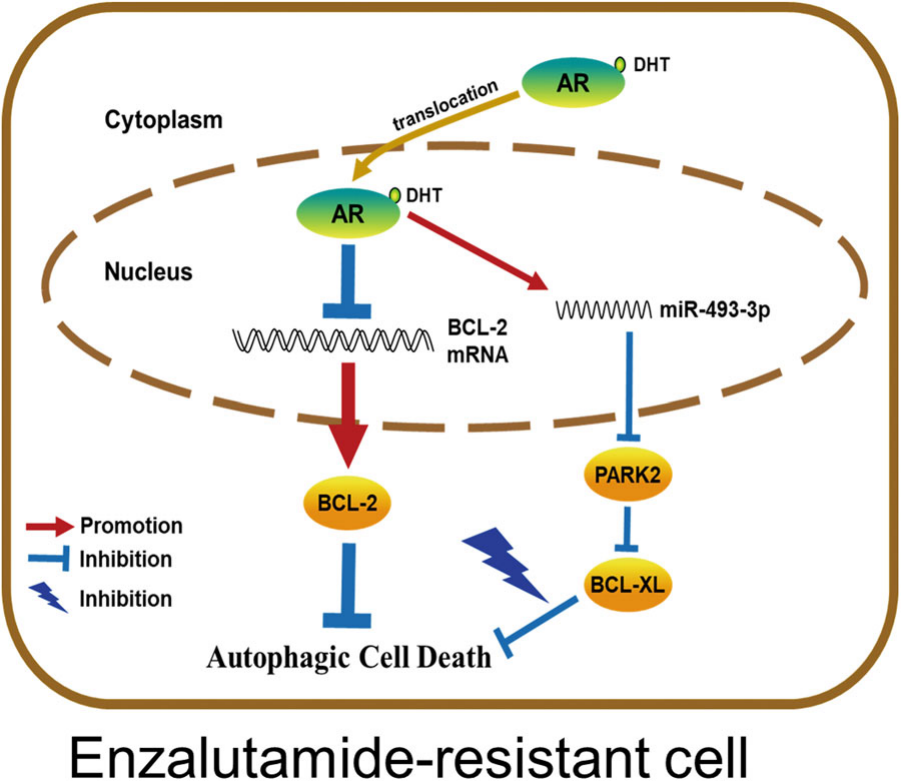
A mechanistic diagram. Mechanistic diagram for DHT effect in CRPC cells.

Together, in vivo results show that suppressing the BCL-XL expression with sh-BCL-XL led to enhance the inhibitory effect of high dose androgen.

## Discussion

PCa bipolar androgen therapy is the process of alternating androgen deprivation followed by androgen activation. Its efficacy has been confirmed recently in some select patients^5,31,32^. As ADT has been effective to suppress PCa cell growth, and has been successfully applied clinically for the treatment of PCa, the notion of high dose DHT to suppress PCa appears to be contradictory to the efficacy of ADT, yet this phenomenon has long been established in xenograft models and human PCa cell lines^13,33,34^. Many mechanisms have been proposed for this finding using several different PCa cells^8^, but so far there is not one cell model that is closer to clinical practice, i.e., to approximate the temporal development of castration-resistant PCa growth followed by inhibition with large doses of androgens. By combining 10 µM Enz and 2 nM DHT in EnzR PCa cells, we found that high doses of DHT (>50 nM), but not a low dose (<10 nM) DHT, could suppress PCa cell growth. It is likely that the low dose DHT cannot counteract the inhibition of Enz while high doses DHT can activate AR through nuclear translocation (Fig. S2A). This system is more closely mimicking the physiological process of bipolar androgen therapy compared to the other system where PCa cells were cultured in charcoal/dextran-stripped fetal bovine serum (CSFBS) medium, which removed not only androgens but also other physiologically important lipid molecules. It was shown that the androgen level in the conventional 1640 medium was lower than the clinical level, and a certain dose of androgen should be added to the culture of PCa cells to simulate the clinical androgen level^35^.

The molecular mechanisms underlying the efficacy of bipolar androgen therapy have been examined before. One hypothesis indicates that AR and topoisomerase 2B may be involved in generating double-stranded DNA breaks, thus responsible for producing genomic rearrangement for the gene fusion of TMPRSS2-ERG. Therefore AR could be involved in DHT-induced PCa inhibition after an increase of DNA damage^11^. Separately it has also been reported that AR can serve as a part of the licensing complex for DNA replication^12,36^ to control cell cycle progression. Hence a higher DHT with activated AR protein can suppress DNA replication and cell proliferation. More recently, it was shown that DHT could inhibit CRPC cells in vivo and in clinical studies^7,10^, likely through cell cycle block through induction of CDK inhibitors, p21 and p27.

Here we find that a high dose androgen can suppress EnzR PCa cells through the induction of autophagic cell death as a result of suppression of BCL-2 expression (Fig. 7). Autophagy can be seen in the body’s physiological and pathological processes. In the normal activities of cells, autophagy is responsible for degrading proteins for cellular homeostasis. In tumor cells, moderate autophagy is the cell’s defense against adverse environments. However, when excessive autophagy occurs, it will cause cell death. This method of death is called autophagic cell death. After endocrine therapy, prostate cancer cells will increase the autophagy to promote cell survival. Therefore, the level of autophagy in EnzR prostate cancer cells is higher. We believe that adding DHT at this time will increase protein synthesis, resulting in excessive accumulation of intracellular proteins, therefore enhanced autophagy and autophagic cell death. Consistent with this, a known autophagy inhibitor, 3-methyadenine (3-MA) could weaken the DHT-induced inhibition of EnzR-PCa cell survival (Fig. S2B). Autophagic cell death is another important death regulation mechanism besides apoptosis. Current studies have found that many tumor suppressor genes and related molecules that regulate autophagy are involved in this process, such as Beclin-1, PTEN, DAPK, p53, and BCL-2^37^. At the same time, cells develop protective mechanisms through enhanced expression of BCL-XL, and suppression of BCL-XL will enhance the death-inducing effect of DHT to slow the progression of CRPC.

The significance of BCL-2 in this process is underscored by the fact that even though high dose DHT represses BCL-2 expression in both DHT-sensitive and DHT-resistant cells (Fig. S2C), the magnitude of BCL-2 reduction is significantly more in cells that are sensitive to DHT than in those that are not. On the other hand, this process was counter balanced by increased expression of BCL-XL due to increased expression of miR-493-3p and consequent repression of BCL-XL E3 ligase PARK2, therefore DHT effect can be enhanced in EnzR cells with a repression of BCL-XL. These results imply that high dose DHT in bipolar androgen therapy may not be effective in PCa patients with balanced apoptotic regulation due to the opposing expression of BCL-2 and BCL-XL. At the same time, it also suggests that bipolar androgen therapy can be enhanced with inhibition of BCL-XL expression. This mechanistic process is consistent with the finding that there is about a threefold increase in the cell death index after androgen treatment in the xenograft model^7^. Moreover, these studies also suggest that the growth inhibition by DHT was not due to a cytostatic effect of supraphysiologic testosterone but rather due to the induction of cell death. Consistent with this, we found that in DHT non-sensitive cells (Enz-S), p21 and p27 proteins were also increased after DHT treatment (data not shown).

The relationship between DHT/AR and anti-apoptotic proteins has been explored before. Many studies have shown that androgens can downregulate the expression of BCL-2 in PCa and breast cancer^15–18^, consistent with that, knocking down AR could increase BCL-2 expression in our system (Fig. S2D). Similarly, it was reported that AR increased the expression of BCL-XL by increasing its mRNA^38^, likely underlying the growth promotion by androgen signaling in PCa. In the EnzR cells, high dose DHT appeared not to regulate BCL-XL transcriptionally but increased its protein expression through a post-transcriptional regulation involving miRNA and E3 ubiquitin ligase (Figs. 3–4). Since BCL-2 and BCL-XL are the members of the same anti-apoptotic protein family, the opposing regulation by DHT of the two proteins appears to cancel each other out, thus cannot be invoked to explain the DHT effect. This notion may only be superficially valid as BCL-2 is not identical to BCL-XL and their opposing change in expression may not result in complete cancelation in function. Indeed, although no classical Caspase-mediated apoptosis was involved (Fig. 5D), autophagic cell death was induced by high dose DHT. More significantly, this mechanistic insight may also point to the potential inherent inefficiency of DHT’s effect on PCa cells due to the internal opposing regulation, and by blocking induction of BCL-XL, high dose DHT may impact more PCa cells, as well as bipolar androgen therapy for more patients.

In summary, our study reveals a novel mechanism for how DHT inhibits CRPC cell growth and potentially new therapeutic approaches to enhance bipolar androgen therapy for patients. Clinical data are needed to fully validate these findings in patients. Nevertheless, these mechanistic insights can potentially help to segregate patients based on BCL-2 and BCL-XL expression, as well as timing for bipolar androgen therapy for the maximal gain of therapeutic efficacy.

## Methods

### Cell culture

Human prostate cancer cell lines LNCAP, C4-2, CWR22Rv-1, PC-3, and DU-145 were obtained from the American Type Culture Collection (ATCC, Rockville, MD) and cultured in RPMI-1640 (without phenol red) supplied with 2 nM DHT, 10% fetal bovine serum (FBS) and 1% Penicillin-Streptomycin, in a 5% (v/v) CO_2_ humidified incubator at 37 °C. EnzR-C4-2 and EnzR-LNCAP cells were generated by culturing EnzS-C4-2 and EnzS-LNCAP cells under increasing Enzalutamide concentrations from 10 μM to 40 μM (every 20 days) for three months. The EnzS-C4-2B and EnzR-C4-2B cells were gifts from Dr. Allen Gao (University of California, Davis, USA). All EnzR cells were maintained in media with 10 µM Enzalutamide.

### Colony-formation assay

Cells were plated in 6-well plates at 200–500 cells/well and attached overnight. EtOH or DHT was added at the second day and cultured at 37 °C, 5% CO_2_. After 10–14 days, colonies were fixed with 4% (w/v) Paraformaldehyde for 15 min and stained with 0.5% Crystal Violet Staining Solution for 20 min for colony visualization.

### RealTime-Glo™ Annexin V assay

The cells were seeded in 96-well plates at a density of 2000 cells/well, adhered overnight, then 50 nM DHT added and cultured at 37 °C, 5% CO_2_ for 48 h. Then, we followed the steps of RealTime-Glo™ Annexin V Apoptosis and Necrosis Assay Kit (Promega) to detect apoptosis and necrosis. EtOH was used as the negative control, and H_2_O_2_ was used as the positive control of 6 h treatment.

### Lentiviral expression plasmids and virus production

The plasmids pLKO-miR-493-3p, pLKO.1-AR, pLKO.1-Beclin-1, pLKO.1-BCL-XL or pWPI-PARK2, pGWB513-BCL-2, the psPAX2 packaging plasmid, and pMD2.G envelope plasmid, were transfected into HEK-293T cells using the standard calcium chloride transfection method for 48 h to get the lentivirus soup. The lentivirus soups were collected and concentrated by density gradient centrifugation and used immediately or frozen at −80 °C for later use.

### RNA extraction and qRT-PCR analysis

For RNA extraction, Trizol reagent (Invitrogen) was used to isolate total RNAs, and 1 µg of total RNA was subjected to reverse transcription into cDNA using Superscript III transcriptase (Invitrogen). Determination of mRNA expression level of an interesting gene was completed using quantitative real-time PCR (qRT-PCR) conducted using a Bio-Rad CFX96 system with SYBR green. The expression of GAPDH was used to normalize the expression levels of a target gene.

### AGO2 immunoprecipitation

The transfected cells were lysed with RIPA lysis buffer (20 nM Tris-HCl (pH 7.5), 1 mM Na_2_EDTA, 150 mM NaCl, 1% NP-40, 1 mM EGTA, 1% sodium deoxycholate, 1 mM beta-glycerophosphate, 2.5 mM sodium pyrophosphate, 1 µg/ml leupetin, 1 mM Na_3_VO_4_,) for 30 mins on ice. Cell suspension was centrifuged at 14,000 rpm for 15 mins. 1/10 supernatant was extracted to detect the concentration of protein to ensure that the input of each group was equal for the next step. The 9/10 supernatant was rotated overnight at 4 °C after adding 10 µl beads and 2 µl AGO2 antibody. The mixture was subsequently washed 3 times with lysis buffer and the RNA was extracted using Trizol reagent (Invitrogen).

### Cell immunofluorescence

The cells were fixed in 4% formaldehyde in PBS for 15 min. The slides were then blocked by 10% horse serum with 0.2% Triton X-100, and co-incubated with primary antibodies (the rabbit anti-AR (Santa Cruz, sc-816), the mouse anti-GAPDH (Santa Cruz, sc-47724)) at 1:100 dilution followed by washing and incubation with secondary antibody and DAPI (Thermo Fisher) for visualization with Nikon Ti-S microscope.

### Immunofluorescence

Cells were transfected with GFP-LC3 plasmid or not, the cells were fixed in 4% formaldehyde in PBS for 10 min followed by permeabilization with 10% Triton X-100. The slides were then blocked by 10% horse serum, and co-incubated with primary antibodies (AR and VHL) at 1:100 dilution followed by washing and incubation with secondary antibody for visualization with confocal microscopy.

### Transmission electron microscopy experiment

Cell samples were processed according to the material requirements (fast, accurate, small), and fixed in 2.5% glutaraldehyde overnight, then rinsed with 0.1 M phosphoric acid rinsing solution followed by 1% osmium acid at 4 °C. The sample was washed with ddH_2_O followed by dehydration and embedding in the embedding template with the addition of pure embedding agent and polymerization in a constant temperature. Semi-thin (1 µm) positioning and ultra-thin microtome sectioning (70 nm) was used for 9.3% uranyl acetate-lead citrate double staining for observation by transmission electron microscope (JEOL, Japan) and then photographed.

### Western blot analysis

Cells were lysed in RIPA buffer and proteins (40 µg) were separated on 8–10% SDS/PAGE gel and then transferred onto PVDF membranes (Millipore). After blocking membranes, they were incubated with appropriate dilutions of specific primary antibodies, and then blots were incubated with HRP-conjugated secondary antibodies and visualized using the ECL system (Thermo Fisher Scientific).

### RNA immunoprecipitation (RIP)

Cells were lysed in ice-cold lysis buffer supplemented with RNase inhibitor. After centrifugation, 10 mg of the supernatant was cleared by protein A/G beads for 1 h and incubated with AGO2 antibody overnight at 4 °C. Then the beads pre-blocked with 15 mg/ml BSA were added to the antibody-lysate mixture and incubated for another 2 h. The RNA/antibody complex was washed four times by RIPA buffer supplemented with RNase inhibitor, protease inhibitor cocktail. The RNA was extracted using Trizol (Invitrogen) according to the manufacturer’s protocol and subjected to qRT-PCR analysis.

### Chromatin immunoprecipitation assay (ChIP)

Normal rabbit IgG (sc-2027, Santa Cruz Biotechnology) and protein A-agarose were used sequentially to preclear the cell lysates. We then added anti-AR antibody (2.0 µg) to the cell lysates and incubated overnight at 4 °C. IgG was used in the reaction for a negative control. Specific primer sets were designed to amplify a target sequence within the human miR-493 promoter, and agarose gel electrophoresis was used to identify the PCR products.

### Luciferase reporter assay

The human 5′-promoter region of miR-493 was constructed into pGL3-basic luciferase reporter vector (Promega). Site-directed mutagenesis of the AR binding site in the miR-493 5′ promoter was achieved with the Quick Change mutagenesis. 2039 bp fragments of PARK2 3′UTR with wild-type or mutant miRNA-responsive elements were cloned into the psiCHECK-2 vector (Promega) downstream of the Renilla luciferase ORF. EnzR-C4-2 and EnzR-C4-2B cells were plated in 24-well plates, and the plasmids were transfected with Lipofectamine 3000 transfection reagent (Invitrogen, Carlsbad, CA) according to the manufacturer’s instructions. PRL-TK was used as an internal control that served as the baseline control response. DHT was added 24 h after transfection. Luciferase activity was measured 48 h after transfection by Dual-Luciferase Assay (Promega) according to the manufacturer’s manual.

### In vivo studies

Due to difficulty of EnzR-C4-2 and EnzR-C4-2B cells to form tumors in mice, EnzR-LNCAP cell was chosen for in vivo studies. 6 weeks old nude mice were purchased from NCI and randomly divided into four groups for injection of 5 × 10^6^ EnzR-LNCAP cells and pre-cultured as follows: 1: pLKO + EtOH; 2: pLKO + Testosterone; 3: sh-BCL-XL + EtOH; and 4: sh-BCL-XL + Testosterone. 5 × 10^6^ EnzR-LNCAP cells were mixed with Matrigel (1:1) and were orthotopiclly transplanted into male Balb/c nude mice. Enz was given through intraperitoneal injection (10 mg/kg/week, twice weekly as shown in Fig. 6A). Bipolar androgen therapy treatment was conducted by injected administration of Testosterone (200 μg/kg/week, twice weekly) or EtOH at the 3rd/5th/7th weeks as shown in Fig. 6A. Mice were sacrificed after 8 weeks, tumors were removed and measured for studies. Each mouse was randomized into each group. A single blind method was performed for analysis. All the animal experiments were performed in accordance with the guidelines of the Tongji University Medical Center Animal Care and Use Committee for animal experiments.

### Statistics

All statistical analyses were carried out with SPSS 19.0 (SPSS Inc, Chicago, IL). The data values were presented as the mean ± SD. Differences in mean values between two groups were analyzed by two-tailed Student’s *t*-test and the mean values of more than two groups were compared with one-way ANOVA. *p* ≤ 0.05 was considered statistically significant.

## Supplementary information

Supplementary figure legends

Supplementary figure 1

Supplementary figure 2

## Data Availability

All the data are available in the article and Supplementary Files, or available from the authors upon request.
