# Supplementary material for: Suppressing BCL-XL increased the high dose androgens therapeutic effect to better induce the Enzalutamide-resistant prostate cancer autophagic cell death
Source: Cell Death Dis. 2021 Jan 11;12(1):68. doi: 10.1038/s41419-020-03321-z (PMC7801470; doi:10.1038/s41419-020-03321-z)
Supplement: Supplementary file 1 — Supplementary figure legends [file 41419_2020_3321_MOESM1_ESM.docx]

**Figure S1 (A)** Formazan formation was increased in the presence of DHT. **(B)** Cell colony formation assay was performed to show that Enzalutamide-resistance (Enz-R) cell growth with 100nM Testosterone. **(C)** Growth of commonly used PCa cell lines under 50nM DHT. **(D)** After sh-BCL-XL transfection, BCL-XL protein expression was reduced. **(E)** Cell colony assay shows that cell growth with 50nM DHT when 10uM Z-VAD-FMK in EnzR-C4-2. **(F)** Percentage of LC3 positive cell increased with 50nM DHT treatment in EnzR-C4-2 cell line transfect EGFP-LC3 plasmid. Data are presented as means ± SD. **p* < 0.05 was considered statistically signiﬁcant by students’ T-test for two groups or ANOVA for more than two groups.

**Figure S2 (A)** Immunofluorescent staining was used on EnzR-C4-2 cell lines to show AR translocation to the nucleus with 50nM DHT treatment for 6 hours. **(B)** Cell colony assay shows that cell growth with 50nM DHT when 1mM 3-MA in EnzR-C4-2. **(C)** Western blotting of BCL-2 expression in different conditions of EnzS-C4-2 and EnzR-C4-2 cells. **(D)** Western blotting showing knocking down AR can increase BCL-2 expression. Data are presented as means ± SD. **p* < 0.05 was considered statistically signiﬁcant by students’ T-test for two groups or ANOVA for more than two groups.
