# Supplementary figures and images for: Suppressing BCL-XL increased the high dose androgens therapeutic effect to better induce the Enzalutamide-resistant prostate cancer autophagic cell death
Source: Cell Death Dis. 2021 Jan 11;12(1):68. doi: 10.1038/s41419-020-03321-z (PMC7801470; doi:10.1038/s41419-020-03321-z)

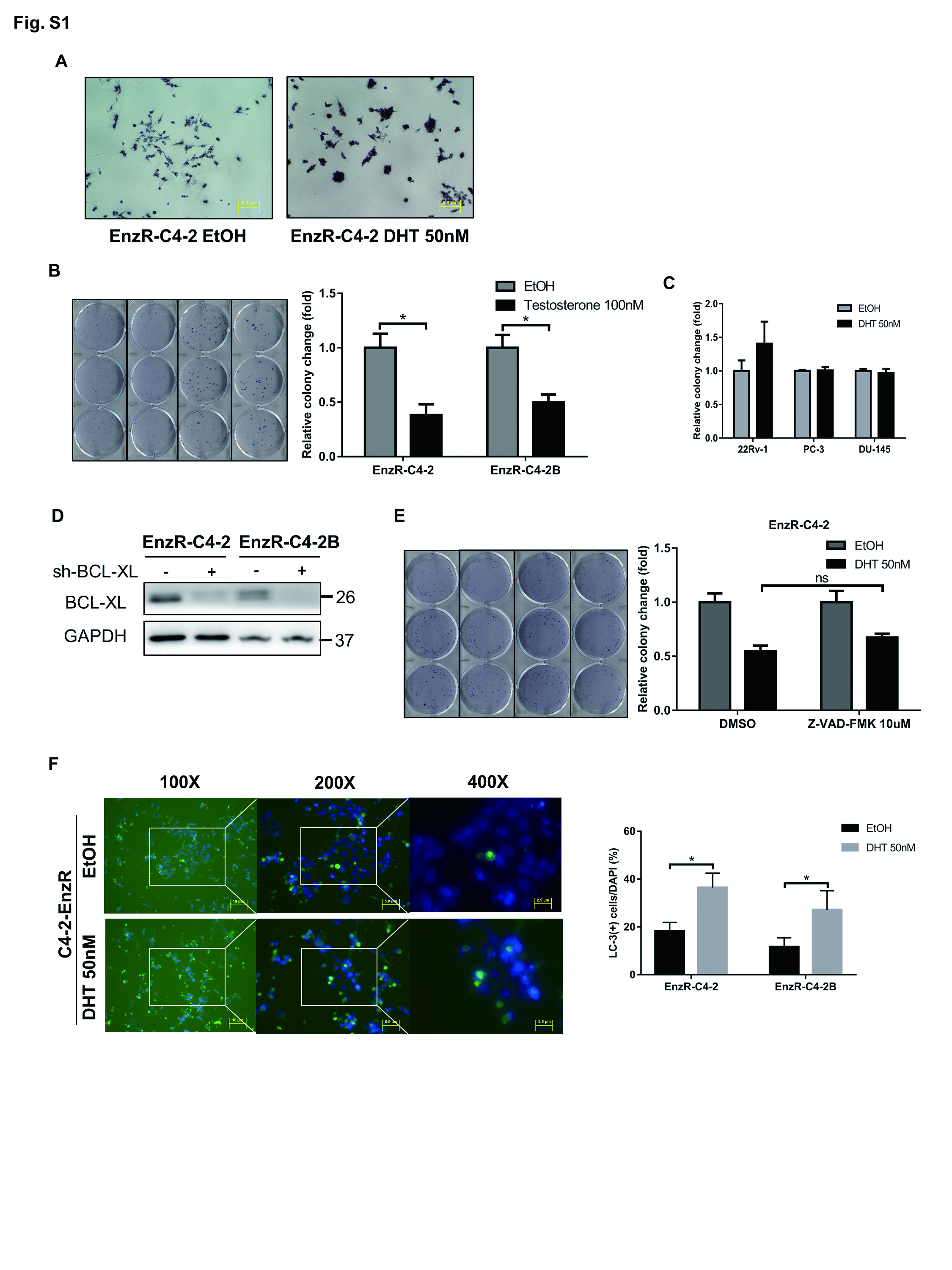

Supplement: Supplementary file 2 — Supplementary figure 1 [file 41419_2020_3321_MOESM2_ESM.tif]

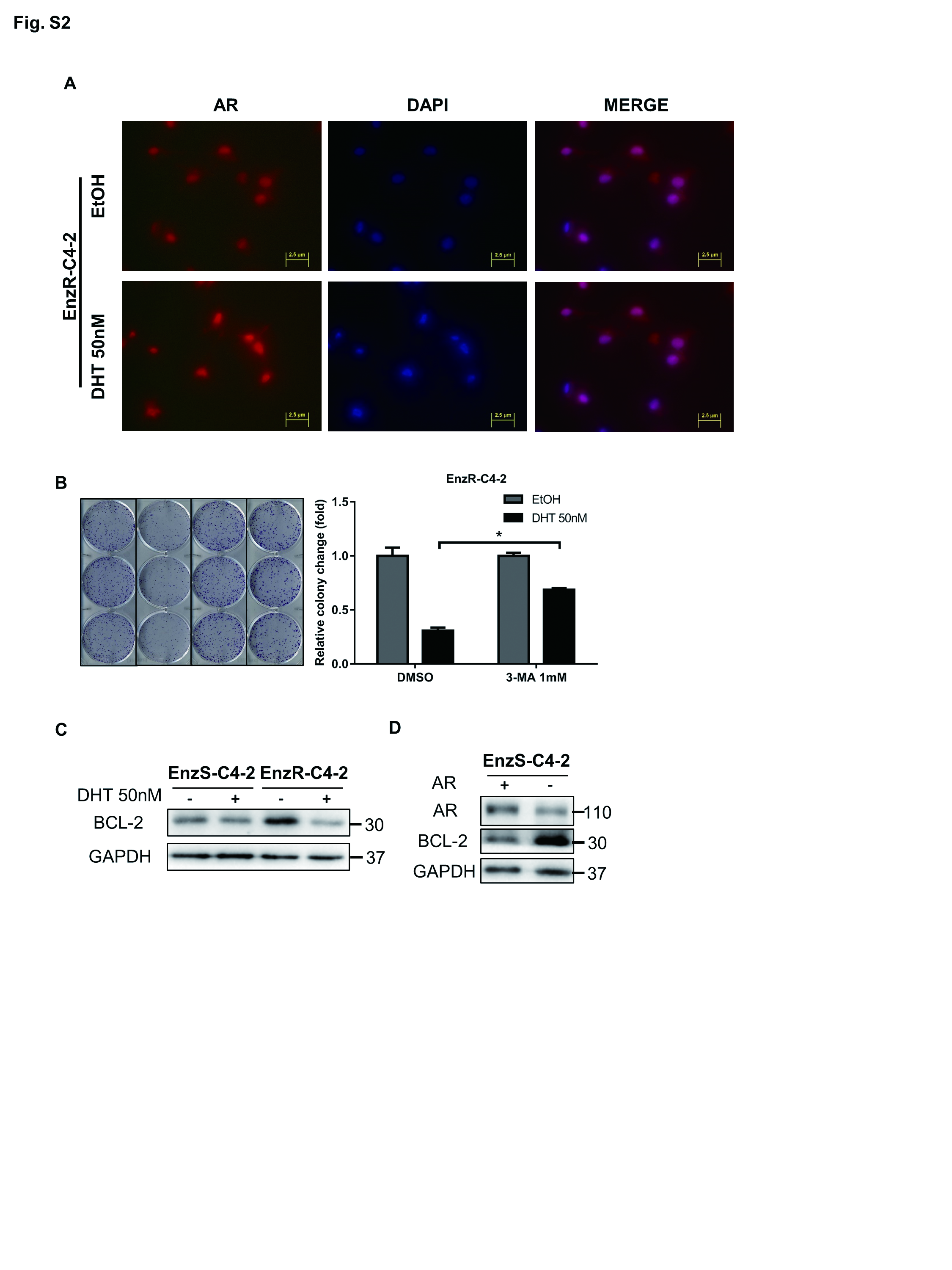

Supplement: Supplementary file 3 — Supplementary figure 2 [file 41419_2020_3321_MOESM3_ESM.tif]
